# Uterine involvement in epithelial ovarian cancer and its risk factors

**DOI:** 10.1186/s13048-021-00925-7

**Published:** 2021-12-07

**Authors:** Narges Zamani, Azam Sadat Mousavi, Setare Akhavan, Shahrzad Sheikhhasani, Somayeh Nikfar, Elham Feizabad, Elahe Rezayof, Mitra Modares Gilani

**Affiliations:** 1 Department of Oncologic Gynecology, Vali-Asr Hospital, Tehran University of Medical Sciences, Tehran, Iran; 2 Department of Obstetrics and Gynecology, Taleghani Hospital, Arak University of Medical Sciences, Arak, Iran; 3 Department of Obstetrics and Gynecology, Yas Hospital, Tehran University of Medical Sciences, Tehran, Iran; 4 Vali-Asr Reproducive Health Research Center, Tehran University of Medical Sciences, Tehran, Iran

**Keywords:** Carcinoma, Ovarian epithelial, Endometrial eeoplasms, Risk eactor

## Abstract

**Background:**

Epithelial ovarian cancer (EOC) is an extremely aggressive and lethal carcinoma. Specific data that identify high-risk groups with uterine involvement are not available. Thus, this study aimed to evaluate a gross number of women with EOC to obtain the frequency of uterine involvement and its risk factors.

**Methods:**

This retrospective observational study was conducted on 1900 histologically confirmed EOC women, diagnosed and treated in our tertiary hospital from March 2009 to September 2020. Data including their demographic, medical and pathological findings were collected.

**Results:**

From 1900 histologically confirmed EOC women, 347 patients were eligible for participations. The mean age of study patients was 51.31 ± 11.37 years with the age range of 25 to 87 years. Uterine involvement was detected in 49.6% (173) of the patients either macroscopic (47.4%) or microscopic (52.6%) types.

Uterine involvement was significantly associated with having AUB (*P*-value = 0.002), histological type of ovary tumor (*P*-value < 0.001), ovarian cancer stage (P-value < 0.001), and abnormal CA-125 concentration (P-value = 0.004).

Compared to the other study patient, the patients with metastatic uterine involvement had significantly higher stage (*p*-value< 0.001), higher grade of ovary tumor (*p*-value = 0.008), serous histological type (p-value< 0.001), and a higher level of CA-125 concentration (*p*-value< 0.001).

on the other hand, the patients with synchronous uterine cancer were significantly younger (*p*-value = 0.013), nulliparous (p-value< 0.001), suffered from AUB symptoms (p-value< 0.001) and had endometroid histological type (p-value = 0.010) of ovary cancer in comparison to other study patients.

**Conclusion:**

Considering the high prevalence of uterine involvement in EOC patients, ultrasound evaluation and/or endometrium biopsy assessment should be done before planning any treatment.

## Background

Epithelial ovarian cancer (EOC) is an extremely aggressive and lethal tumor, mostly diagnosed in advanced stages with poor prognosis except in small number of patients with early detection [1].

Nearly 10% of all patients with EOC seem to have concurrent endometrial cancer, which are the most common synchronous gynecologic tumors. Evidentially, concurrent uterine involvement in EOC patients is mostly observed in younger nulliparous women with lower ovary tumor stage [1–3].

It is so important to apply a comprehensive strategy in the diagnosis and treatment of this tumor [4]. The routine therapeutic approach for EOC management is primary debulking or neoadjuvant chemotherapy followed by surgical cytoreduction [5, 6].

Not surprisingly, hysterectomy is usually used in primary debulking surgery in advanced invasive EOC management. Although there is no clear logical reason, at least, preoperative recognition of patients according to uterine involvement could be helpful for selecting hysterectomy type (total or subtotal) [7–10].

On the other hand, since the most important prognostic factor in EOC patients is the residual tumor measures, it seems, saving cancer-free uterine, as well as other intraperitoneal organ maintenance with no cancer evidence, does not have any adverse impact on the patient prognosis [11].

In contrast, the most reason for hysterectomy include a large number of EOC patients with uterine involvement and the improvement of the overall survival rate in high-risk patients [5, 6]. Additionally, no specific data are available to identify high-risk groups for concurrent endometrial cancer [12, 13].

To the best of our knowledge, the strong evidence about the frequency of uterine involvement and its related causes is limited [11]. Thus, this study aimed to evaluate a gross number of women with EOC to obtain the frequency of uterine involvement and its risk factors.

## Materials and methods

This retrospective observational study was conducted on histologically confirmed EOC women, diagnosed and treated in Imam Khomeini hospital from March, 2009 to September, 2020.

From among all histologically confirmed EOC women who were referred to our oncology department, the ones on whom hysterectomy as a debulking primary surgery was performed, enrolled in the study.

The patients with borderline or non-epithelial ovarian tumors, neoadjuvant chemotherapy, and uterine maintenance during primary surgery were excluded. In addition, uterine cancer patients with metastasis to ovary as well as those whose medical records were missing were excluded from the study.

Cytoreduction surgery in our center includes total abdominal hysterectomy (TAH), bilateral salpingo-oophorectomy (BSO), omentectomy, bilateral pelvic lymph node dissection, peritoneal biopsy, peritoneal fluid cytology study and appendectomy.

All materials were examined in the pathology laboratory of our hospital. The uterine involvement diagnosis was done according surgical and pathological findings.

After surgery, six sessions of chemotherapy (Paclitaxel and Carboplatin) were done each 21 days for the patients if needed.

Medical information such as age, menopausal state, obstetric history, AUB symptom, International Federation of Gynecology and Obstetrics (FIGO) stage scoring, pathology type, the concurrent involvement of other organs, tumor marker concentration (CA125, and HE4) were collected.

### Statistical analysis

All the data were analyzed using SPSS version 24.0 (IBM, New York, USA). A *P*-value of less than 0.05 was determined as the level of statistical significance. We used Independent T-test and Non-parametric Mann–Whitney U-test to assess differences in means. We applied the chi-square test to evaluate the proportional differences.

## Results

From 1900 histologically confirmed EOC women, 347 patients were eligible for participations. The mean age of study patients was 51.31 ± 11.37 years with the age range of 25 to 87 years. Of them, 53% were post-menopausal and 17% were nulliparous. AUB was reported in 8.1% of them.

At the time of disease detection, 51.3% (178) of the participants had stage III of EOC and about 68% of them were of serousal histology type. High-grade tumors were reported in 48.7% (169) of them.

**Fig. 1:**
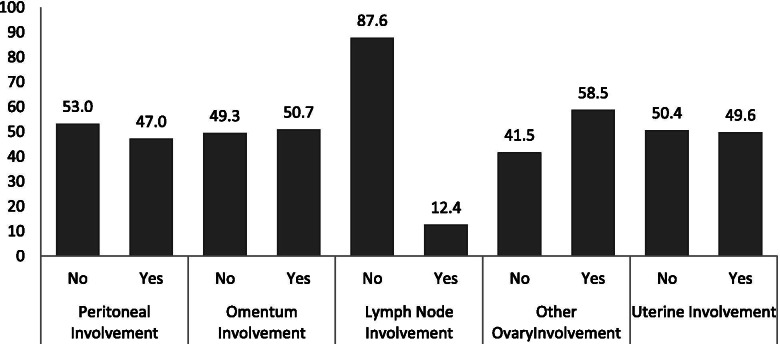
The percent of organ involvement in patients with epithelial ovarian cancer

The most common concurrent organ involvement (58.5%) was reported in the other patient’s ovary. Uterine involvement was detected in 49.6% (173) of the patients either macroscopic (47.4%) or microscopic (52.6%) types. Other organ involvements is shown in Fig. 1.

**Fig. 2:**
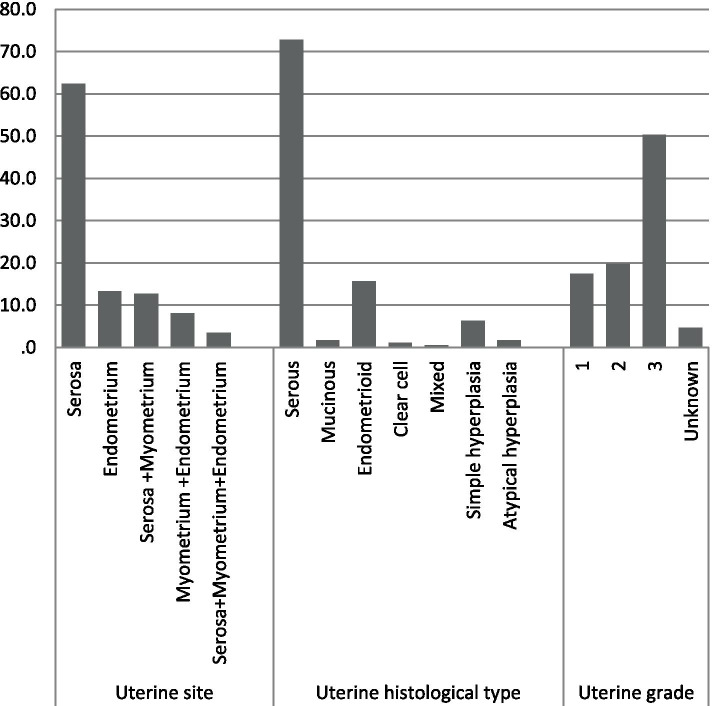
The involvement of uterine sites

In patients with uterine involvement, serosal histology (72.8%), the involvement of serous layer (83.2%) and grade III (50.3%) of uterine tumors were the most prevalent. Isolated endometrium involvement was present only in 23 women (13.3%) (Fig. 2).

Furthermore, our study showed that uterine involvement was significantly associated with having AUB (*P*-value = 0.002), histological type of ovary tumor (P-value < 0.001), ovarian cancer stage (P-value < 0.001), and abnormal CA-125 concentration (P-value = 0.004) (Table 1).

**Table 1:** The distribution of selected demographic and tumor related characteristics

Characteristics	Overall	Uterine involvement		***P***-value
		No (***N*** = 174)	Yes (***N*** = 173)	
**Age**	51.31 ± 11.37	51.01 ± 11.36	51.61 ± 11.41	0.620
**Nulliparous**	59 (17)	28 (16)	31 (17.9)	0.651
**Post-menopausal**	184 (53)	91 (52.2)	93 (53.7)	0.785
**AUB**	28 (8.1)	6 (3.4)	22 (12.7)	0.002
**Histological type of OC**				
Serous	235 (67.7)	96 (55.1)	139 (80.3)	< 0.001
Mucinous	21 (6.1)	16 (9.2)	5 (2.9)	
Endometrioid	61 (17.6)	37 (21.2)	24 (13.9)	
Clear cell	20 (5.8)	17 (9.8)	3 (1.7)	
Unknown	2 (0.6)	1 (0.6)	1 (0.6)	
Mixed	8 (2.3)	7 (4)	1 (0.6)	
**OC stage**				
1	119 (34.3)	98 (56.3)	21 (12.1)	< 0.001
2	42 (12.1)	21 (12.1)	21 (12.1)	
3	178 (51.3)	55 (31.6)	123 (72)	
4	8 (2.3)	0	8 (4.6)	
**OC Grade**				
1	82 (23.6)	50 (28.7)	32 (18.5)	0.070
2	76 (21.9)	33 (19)	43 (25)	
3	169 (48.7)	84 (48.2)	85 (49)	
Unknown	20 (5.8)	7 (4.1)	13 (7.5)	
**CA-125**^a^				
< 35	32 (9.2)	23 (13.2)	9 (5.2)	0.004
> 35	273 (78.7)	122 (70.1)	151 (87.2)	

^a^42 missing data: In patients without uterine involvement, 29 patients and in uterine involvement group, 13 patients with missing data on CA-125

To do a more accurate analysis, the patients with uterine involvement were categorized into two groups: metastatic (136) and synchronous endometrial disorder (37) types. Compared to the patients in other studies, the patients with metastatic uterine involvement showed no significant distinction in regard to age, menopausal status, parity, and AUB symptom. However, the patients with metastatic uterine involvement had significantly higher stage (*p*-value< 0.001), higher grade of ovary tumor (*p*-value = 0.008), serous histological type (*p*-value< 0.001), and a higher level of CA-125 concentration (*p*-value< 0.001) (Table 2).

**Table 2:** The comparison of selected demographic and tumor related characteristics between metastatic and synchronous endometrial disorder and other epithelial ovarian cancer

Characteristics	Metastatic (***N*** = 136)	Others (***N*** = 211)	***P***-value^a^	Synchronous (***N*** = 37)	***P***-value^b^
**Age, yrs.**	52.32 ± 11.24	50.65 ± 11.43	0.182	49 ± 11.78	0.117
**Pre-menopausal**	60 (44.1)	103 (48.8)	0.392	20 (54.1)	0.282
**Post-menopausal**	76 (55.9)	108 (51.2)		17 (45.9)	
**Nulliparous**	20 (14.7)	39 (18.5)	0.360	11 (29.7)	0.035
**Multiparous**	116 (85.3)	172 (81.5)		26 (70.3)	
**Without AUB**	126 (92.6)	193 (91.5)	0.694	25 (67.6)	< 0.001
**With AUB**	10 (7.4)	18 (8.5)		12 (32.4)	
**Histological ovary type**					
**Serous**	124 (91.2)	111 (52.6)	< 0.001	15 (40.5)	< 0.001
**Mucinous**	3 (2.2)	18 (8.5)		2 (5.4)	
**Endometrioid**	7 (5.1)	54 (25.6)		17 (45.9)	
**Clear cell**	1 (0.7)	19 (9)		2 (5.4)	
**Unknown**	0	2 (0.9)		1 (2.7)	
**Mixed**	1 (0.7)	7 (3.3)		0	
**Ovary tumor stage**					
**1**	0	119 (56.4)	< 0.001	21 (56.8)	< 0.001
**2**	19 (14)	23 (10.9)		2 (5.4)	
**3**	109 (80.1)	69 (32.7)		14 (37.8)	
**4**	8 (5.9)	0		0	
**Ovary tumor grade**					
**1**	19 (14)	63 (29.9)	0.008	13 (35.1)	0.003
**2**	32 (23.5)	44 (20.9)		11 (29.7)	
**3**	76 (55.9)	93 (44.1)		9 (24.3)	
**Unknown**	9 (6.6)	11 (5.2)		4 (10.8)	
**CA125**					
**< 35**	6 (4.7)	26 (14.7)	0.005	3 (9.4)	0.303
**> 35**	122 (95.3)	151 (85.3)		29 (90.6)	
**CA125**					
**< 100**	22 (17.2)	66 (37.3)	< 0.001	11 (34.4)	0.032
**> 100**	106 (82.8)	111 (62.7)		21 (65.6)	

^a^metastatic uterine involvement and other epithelial ovarian cancer comparison, ^b^ metastatic uterine involvement and synchronous endometrial disorder comparison

In contrast, the patients with synchronous uterine cancer were significantly younger (p-value = 0.013), nulliparous (p-value< 0.001), suffered from AUB symptoms (p-value< 0.001) and had endometroid histological type (p-value = 0.010) of ovary cancer in comparison to other study patients (Table 3).

**Table 3:** The comparison of selected demographic and tumor related characteristics between synchronous endometrial cancer (synchronous endometrial disorder without simple hyperplasia) and other epithelial ovarian cancer

Characteristics	Others (***N*** = 321)	Synchronous without simple hyperplasia (***N*** = 26)	***P***-value
**Age**	51.74 ± 11.4	46.00 ± 9.76	0.013
**Pre-menopausal**	147 (45.8)	16 (61.5)	0.122
**Post-menopausal**	174 (54.2)	10 (38.5)	
**Nulliparous**	48 (15)	11 (42.3)	< 0.001
**Multiparous**	273 (85)	15 (57.7)	
**Without AUB**	303 (94.4)	16 (61.5)	< 0.001
**With AUB**	18 (5.6)	10 (38.5)	
**Histological type ovary**			
Serous	225 (70.1)	10 (38.5)	0.010
Mucinous	20 (6.2)	1 (3.8)	
Endometrioid	48 (15)	13 (50)	
Clear cell	19 (5.9)	1 (3.8)	
Unknown	1 (0.3)	1 (3.8)	
Mixed	8 (2.5)	0	
**ovary stage**			
1	102 (31.8)	17 (65.4)	0.002
2	41 (12.8)	1 (3.8)	
3	170 (53)	8 (30.8)	
4	8 (2.5)	0	
**Grade ovary**			
1	71 (22.1)	11 (42.3)	0.006
2	71 (22.1)	5 (19.2)	
3	163 (50.8)	6 (23.1)	
Unknown	16 (5)	4 (15.4)	
**CA125**			
< 35	30 (10.6)	2 (9.1)	0.824
> 35	253.3 (89.4)	20 (90.9)	
**CA125**			
< 100	82 (29)	6 (27.3)	0.865
> 100	201 (71)	16 (72.7)	

## Discussion

Ovary cancer (OC) is one of the most common lethal and aggressive gynecologic cancers. The traditional treatment of OC involves resecting all suspected organs followed by chemotherapy. However, nowadays, it is preferred to use conservative surgery, especially in young patients aiming at fertility preservation [11].

In ovarian cancer, serous carcinoma was the most common histologic type and most of the patients had advanced (high stage and grade) disease at presentation. Our findings were in line with Dvoretsky el study in terms of commonly histological type, stage, and grade distribution of ovarian cancer [14].

It is so critical to know about uterine involvement in EOC patients before or at least during surgery because it would affect clinical management, prognosis, and surgeons’ decision for whether hysterectomy is required or not, and if it is required, a total or subtotal hysterectomy should be done [15]. However, information about uterine involvement in EOC is rare, and the best management approaches have not been evaluated in enough reviews [8–10].

To the best of our knowledge, for the first time in this study, a relatively high sample size of EOC patients either without or with uterine involvement (metastatic or synchronous) assessed to gather some valuable information on this issue.

As this study showed, high-grade serous ovarian carcinoma was the most common histological ovary type in metastatic uterine involvement in comparison to endometroid type that is the most prevalent in synchronous ovarian and uterine carcinoma. It is worth mentioning, metastatic uterine carcinoma negatively changes both the patient’s prognosis and treatment and increases recurrence and death chances [16, 17].

In Menczer et al’s study, uterine involvement was reported in 52.5%, mostly in high stage and grade EOC patients and it was macroscopic only in 14.1% of them. The serosal layer of the uterine was the most common site of involvement [18]. In accordance with Menczer et al’ study, uterine involvement was detected in 49.6% of our participants with mostly in a serosal layer of uterine, however, the frequency of macroscopic type was 47.4% in our study.

In contrast with our study, the study byKitratara et al. indicated that the frequency of uterine involvement was not common (18.4%), which often was detected in high grade and macroscopic involvement type of EOC without any relation to disease stage and histology [11].

Bunting et al. study on ovarian cancer patients indicated that hysterectomy itself does not determine the patient prognosis, however, the post-operation residual tumor size is the most important factor in prognosis. Further studies are needed about the hysterectomy effect on the patients’ survival rate [19].

In our study, the metastatic uterine involvement patients had higher age and are more frequent in menopausal status. About 85% of them were multiparous and AUB was reported only in 7.4%. Also, they had a higher stage (80.1% in stage III) and grade of disease with the serousal histological type as the most common type. The tumor markers, against the former studies, were evaluated in this study. It is worth mentioning, CA-125 was great than 100 in 82.8% of metastatic uterine involvement patients.

In former studies, the frequency of synchronous uterine involvement in OC ranged 0.8 to 10% [1, 11, 15, 20, 21]. This wide range might be due to different targeted study populations, for instance, this prevalence was higher when patients with OC were the study sample and it was less in the endometrial cancer sampling study. This prevalence was 10.6% in our study, while this was 7.5% according to Stocully et al. study [22] and considering only endometrial cancer and atypical endometrial hyperplasia (without considering simple hyperplasia).

As our study showed, synchronous ovarian and uterine carcinoma reported in patients with the low stages of the disease, and these women have an excellent prognosis with a survival rate of 80 to 90% [16]. Furthermore, the study patients with synchronous endometrial cancer were significantly younger (mean age: 46 years), mostly pre-menopausal (61%), 42% of them were nulliparous, which was in accordance with former studies [23–25].

As mentioned, patients with synchronous endometrial cancer are often young and nulliparous and willing to maintain their fertility ability, but if the synchronous endometrial disorder was diagnosed in these patients, we must do the hysterectomy. On the other hand, genetic disorders are probable, because of the tumor presentation onset at a lower age, although further research is needed in this thesis.

AUB symptoms were reported in 61% of patients with synchronous endometrial cancer, while this was only in 5% of other patients. In fact, the most common chief complaint in these patients is AUB [13, 15, 23–27].

Similar to this study, endometrioid histology is the most common histology in patients with synchronous endometrial cancer; mostly there are low stage and grade of diseases in these patients [13, 15, 24–28].

CA-125 concentration was significantly higher in metastatic uterine involvement rather than synchronous endometrial cancer, while our finding was in line with Broeders et al. study [23], few studies were on this topic. In addition, Due to a large number of participants with a lack of HE4 assessment, this tumor marker could not be compared between different study groups.

This study, similar with the previous studies, showed that patients with synchronous endometrial cancer are mostly younger, nulliparous, having AUB symptoms, with lower stage and grade of disease, endometrioid histology, and lower CA-125 concentration.

With regards to the fertility maintenance request in these patients, endometrial evaluation with ultrasound or D&C biopsy must be done first to decide about the uterine saving or hysterectomy (subtotal or total) type selection.

On the other, the frequency of the synchronous endometrial disorder in EOC is considerable even in the absence of AUB symptoms or endometrioid histology, in lower stage and grade of disease, so it seems endometrial evaluation with ultrasound or D&C biopsy should be performed before any treatment decisions.

This study had some limitations. The study was done retrospectively and some other variables, including residual disease, immuno-histochemical reports, and HE4 tumor markers could not be evaluated because their data was not available.

It seems that there is a growing need for further research in concurrent ovarian and endometrial carcinoma in both biological and clinical topics. In addition, further research is recommended for comparing uterine involvement and the overall survival in EOC patients with and without hysterectomy.

## Conclusion

Nowadays, it is preferred to have a conservative and less invasive surgery therapy in all gynecological cancers, as well as, in ovarian cancers, the uterine can be saved even in the higher stage of disease after rule out any synchronous endometrial disorder.

Considering the high prevalence of uterine involvement in EOC patients, ultrasound evaluation and/or endometrium biopsy assessment should be done before any treatment planning, especially in patients who demand to preserve their fertility.

## Data Availability

The datasets used and/or analyzed in this study are available from the corresponding author on reasonable request.

## References

[1] Chiang Y-C, Chen Y-C, Huang C-Y, Hsieh C-Y, Cheng W-F (2008). Synchronous primary cancers of the endometrium and ovary. Int J Gynecol Cancer.

[2] Soliman PT, Slomovitz BM, Broaddus RR, Sun CC, Oh JC, Eifel PJ (2004). Synchronous primary cancers of the endometrium and ovary: a single institution review of 84 cases. Gynecol Oncol.

[3] Zaino R, Whitney C, Brady MF, DeGeest K, Burger RA, Buller RE (2001). Simultaneously detected endometrial and ovarian carcinomas. A prospective clinicopathologic study of 74 cases: a Gynaecologic oncology group study. Gynecol Oncol.

[4] Kuroki L, Guntupalli SR (2020). Treatment of epithelial ovarian cancer. BMJ..

[5] Bafghi A, Zafrani Y, Pautier P (2007). Endometrial disorders in patients with peritoneal serous papillary carcinoma. Eur J Obstet Gynecol Reprod Biol.

[6] du Bois A, Reuss A, Pujade-Lauraine E (2009). Role of surgical outcome as prognostic factor in advanced epithelial ovarian cancer: a combined exploratory analysis of 3 prospectively randomized phase 3 multicenter trials: by the Arbeitsgemeinschaft Gynaekologische Onkologie Studiengruppe Ovarialkarzinom (AGO-OVAR) and the Groupe d’Investigateurs Nationaux pour les etudes des cancers de l’Ovaire (GINECO). Cancer..

[7] Marsden DE, Friedlander M, Hacker NF (2000). Current management of epithelial ovarian carcinoma: a review. Semin Surg Oncol.

[8] Trope C, Kaern J (2006). Primary surgery for ovarian cancer. Eur J Surg Oncol.

[9] Leitao MM, Chi DS (2007). Operative management of primary epithelial ovarian cancer. Curr Oncol Rep.

[10] Fader AN, Rose PG (2007). Role of surgery in ovarian carcinoma. J Clin Oncol.

[11] Chitrathara K, Sheikh ZA, Vijaykumar DK, Kuriakose S, Anupama R, Nandeesh M (2011). Is hysterectomy needed in ovarian cancer?. Indian J Cancer.

[12] Roth LM, Emerson RE, Ulbright TM (2003). Ovarian endometrioid tumors of low malignant potential: a clinicopathologic study of 30 cases with comparison to well-differentiated endometrioid adenocarcinoma. Am J Surg Pathol.

[13] Jia SZ, Zhang JJ, Yang JJ, Xiang Y, Liang Z, Leng JH (2018). Risk of synchronous endometrial disorders in women with endometrioid borderline tumors of the ovary. J Ovarian Res.

[14] Dvoretsky PM, Richards KA, Angel C, Rabinowitz L, Stoler MH, Beecham JB (1988). Distribution of disease at autopsy in 100 women with ovarian cancer. Hum Pathol.

[15] Williams MG, Bandera EV, Demissie K, Rodríguez-Rodríguez L (2009). Synchronous primary ovarian and endometrial cancers: a population-based assessment of survival. Obstet Gynecol.

[16] Dizon DS, Birrer MJ (2016). Making a difference: distinguishing two primaries from metastasis in synchronous tumors of the ovary and uterus. J Natl Cancer Inst.

[17] Wu RC, Veras E, Lin J, Gerry E, Bahadirli-Talbott A, Baras A, Ayhan A, Shih IM, Wang TL (2017). Elucidating the pathogenesis of synchronous and metachronous tumors in a woman with endometrioid carcinomas using a whole-exome sequencing approach. Cold Spring Harb Mol Case Stud.

[18] Menczer J, Chetrit A, Sadetzki S (2010). National Israel Ovarian Cancer Group. Uterine metastases in ovarian carcinoma: frequency and survival in women who underwent hysterectomy. J Gynecol Oncol.

[19] Bunting MW, Jaaback KS, McNally OM (2011). Routine hysterectomy in the surgical management of ovarian cancer: a retrospective case series, physician opinion survey, and review of the literature. Int J Gynecol Cancer.

[20] Zaino R, Whitney C, Brady MF, DeGeest K, Burger RA, Buller RE (2001). Simultaneously detected endometrial and ovarian carcinomas–a prospective clinicopathologic study of 74 cases: a gynecologic oncology group study. Gynecol Oncol.

[21] van Niekerk CC, Vooijs GP, Bulten J, van Dijck JA, Verbeek AL (2007). Increased risk of concurrent primary malignancies in patients diagnosed with a primary malignant epithelial ovarian tumor. Mod Pathol.

[22] Scully RE, Young RH, Clement PB (1998). Tumors of the ovary, Maldeveloped gonads, fallopian tube, and broad ligament.

[23] Broeders FM, van der Wurff AA, Pijnenborg JM, Vos MC (2012). Preoperative identification of synchronous ovarian and endometrial cancers: the importance of appropriate workup. Int J Gynecol Cancer.

[24] Rodolakis A, Thomakos N, Akrivos N, Sotiropoulou M, Ioannidis I, Haidopoulos D, Vlachos G, Antsaklis A (2012). Clinicopathologic insight of simultaneously detected primary endometrial and ovarian carcinomas. Arch Gynecol Obstet.

[25] Sozen H, Vatansever D, Iyibozkurt AC, Topuz S, Ozsurmeli M, Salihoglu Y, Guzelbey B, Berkman S (2015). Clinicopathologic and survival analyses of synchronous primary endometrial and epithelial ovarian cancers. J Obstet Gynaecol Res.

[26] Solmaz U, Karatasli V, Mat E, Dereli L, Hasdemir PS, Ekin A, Gezer C, Sayhan S, Sanci M, Guvenal T (2016). Synchronous primary endometrial and ovarian cancers: a multicenter review of 63 cases. Tumori..

[27] Narin MA, Karalok A, Basaran D, Ureyen I, Turkmen O, Turan T, Tulunay G (2015). Coexisting primary cancers of endometrium and ovary: experience of single institution. Int J Hematol Oncol.

[28] van Niekerk CC, Bulten J, Vooijs GP, Verbeek AL (2010). The association between primary Endometrioid carcinoma of the ovary and synchronous malignancy of the endometrium. Obstet Gynecol Int.

